# Subgingival Use of Air-Polishing Powders: Status of Knowledge: A Systematic Review

**DOI:** 10.3390/jcm12216936

**Published:** 2023-11-05

**Authors:** Dorin Nicolae Gheorghe, Francesco Bennardo, Margarita Silaghi, Dora-Maria Popescu, George-Alexandru Maftei, Marilena Bătăiosu, Petra Surlin

**Affiliations:** 1 Department of Periodontology, Research Center of Periodontal-Systemic Interactions, Faculty of Dental Medicine, University of Medicine and Pharmacy of Craiova, 200349 Craiova, Romania; popescu131@yahoo.com (D.-M.P.); surlinpetra@gmail.com (P.S.); 2 Department of Health Sciences, School of Dentistry, Magna Graecia University of Catanzaro, 88100 Catanzaro, Italy; 3 Faculty of Dental Medicine, University of Medicine and Pharmacy of Craiova, 200349 Craiova, Romania; margaritasil@outlook.com.gr; 4 Department of Dento-Alveolar Surgery and Oral Pathology, “Grigore T. Popa” University of Medicine and Pharmacy, 700115 Iași, Romania; george-alexandru.maftei@umfiasi.ro; 5 Department of Pedodontics, University of Medicine and Pharmacy of Craiova, 200349 Craiova, Romania; marilena.bataiosu@umfcv.ro

**Keywords:** periodontal, air-polishing, subgingival, periodontal therapy, glycine, erythritol

## Abstract

Effective subgingival biofilm removal is crucial for achieving positive and stable outcomes in periodontal therapy, forming an indispensable part of any periodontal treatment approach. The development of air-polishing tools has emerged as a promising alternative to hand and ultrasonic scalers for dental biofilm removal. The objective of this systematic review was to assess existing literature regarding the subgingival use of various types of air-polishing powders, as an effective method of subgingival biofilm control. For this, 55 articles on this subjected were sourced from searched databases and subjected to an evaluation process of their contained information, which was subsequently structured and compiled into this manuscript. The existing literature acknowledges that good subgingival biofilm control is essential for the success of periodontal therapy, including through subgingival air-polishing, as an adjunctive procedure. This approach has the potential to enhance patient comfort during and after subgingival mechanical plaque removal, thereby mitigating damage to periodontal structures. Consequently, it may lead to improved healing capabilities within the periodontal tissues and the formation of a more stable reparative gingival junctional epithelium.

## 1. Introduction

Periodontitis is a chronic and multifactorial inflammatory condition characterized by the gradual loss of teeth-supporting structures, including alveolar bone and the periodontal ligament, resulting from the accumulation of dental plaque or biofilm [1]. Typical clinical signs of periodontitis include gingival inflammation, radiographic evidence of alveolar bone loss, clinical attachment loss, deep probing depths, bleeding on probing, mobility, and pathologic migration [2]. The staging system for periodontitis categorizes the disease into four stages (stage I, stage II, stage III, and IV) based on the severity, complexity, extent, and distribution of the disease, as determined based on clinical attachment loss, radiographic bone loss, and tooth loss [3]. The grading system (grade A, grade B, grade C) reflects the biological aspects of the infection, such as indicators or risks of rapid disease progression, anticipated treatment outcomes, and consequences for systemic health [4]. The development of this new classification system has been the first step towards the implementation of a pan-European clinical guideline for the treatment of stage 1–3 and stage 4 periodontitis [3]. Thus, the guideline issued by the European Federation of Periodontology offers clinicians valuable recommendations for the treatment of periodontitis, using both non-surgical and surgical means. Despite the different treatment options, the guideline emphasizes the paramount role of efficient biofilm control [4]. Consequently, this could be achieved through the subgingival use of air-polishing, as an adjunctive procedure to subgingival instrumentation. 

The primary causative factor of periodontitis is the accumulation of dental biofilm. However, the disease pathogenesis is multifactorial and involves complex interactions among specific bacterial infections (red-complex bacteria, including *Porphyromonas gingivalis*, *Tannerella forsythia*, and *Treponema denticola*, which are predominantly found in deep periodontal pockets), dysregulated host immune responses, and environmental factors, such as smoking [5]. These interrelated factors contribute to the progression of the disease through intricate and dynamic interactions [6].

Smoking represents the most significant environmental risk factor for periodontitis. Individuals who smoke exhibit a higher prevalence and quantity of red-complex periodontal bacteria within their subgingival biofilm when compared to nonsmokers or former smokers [7,8]. Additionally, smoking has been implicated in the impairment of host immune cell function, particularly that of neutrophils, which increases susceptibility to periodontitis [9]. Both active periodontal therapy and long-term maintenance periodontal therapy are affected by the adverse effects of smoking. Therefore, it is crucial for patients to be continually reminded of the importance of smoking cessation in achieving effective periodontitis care [10].

The development of air-polishing tools has emerged as a viable alternative to hand and ultrasonic scalers for the removal of dental biofilm [11,12]. These tools function by projecting a stream of compressed air, mixed with water and abrasive particles such as sodium bicarbonate, glycine, trehalose, and erythritol, onto the tooth surface, effectively eliminating the biofilm [13,14]. Unlike hand and ultrasonic instruments, air-polishing equipment only removes biofilm, reducing clinical time and causing less discomfort to patients. Air-polishing can be used alone or in combination with hand instrumentation to eliminate residual pockets during initial or supportive periodontal therapy [15]. 

The food industry has embraced erythritol, an alcohol sugar, as an artificial sweetener [16]. This sugar substitute is not metabolized after consumption and is excreted intact in urine, making it a safe daily dietary option [17,18]. Erythritol has a sweet taste, which makes it safe for use in the oral cavity, and it is well-tolerated by individuals [18]. Additionally, erythritol is not cariogenic, which means that it does not contribute to tooth decay [19,20]. Although erythritol has limitations in removing large and firmly attached deposits of calculus and other hard substances, it is a suitable alternative to supportive periodontal therapy when used as an adjuvant to active periodontal therapy [21,22]. Moreover, erythritol exhibits antimicrobial properties that inhibit the growth of periodontal infections, as suggested by research [23]. 

The objective of this review was to address three main questions. Firstly, the review aimed to determine whether subgingival air-polishing is a viable alternative to ultrasonic scalers or whether it is more effective when used in combination with other tools. Secondly, the review sought to identify the most effective and least traumatic airflow powder for subgingival air-polishing. Finally, the review aimed to assess whether subgingival air-polishing can be safely used on both natural teeth and implant structures.

## 2. Materials and Methods

**Figure 1:**
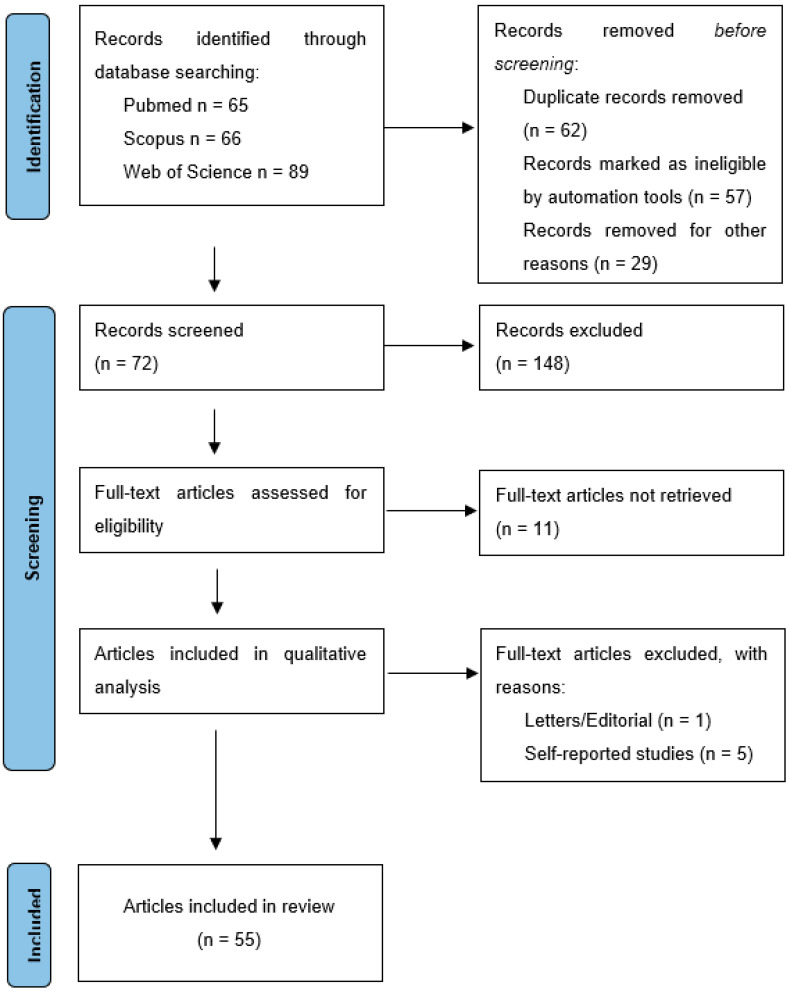
PRISMA flow diagram.

This systematic review adhered to the Preferred Reporting Items for Systematic Review and Meta-Analyses (PRISMA) guidelines and followed a PICO framework to answer the research questions (Figure 1). 

### 2.1. PICO Questions

The PICO question for this systematic review is as follows: “In patients with periodontal disease, can professional-mechanical plaque removal with sub-gingival air-polishing have the same efficiency as ultrasonic devices for effective control of subgingival biofilm deposits during periodontal therapy?” (Population: patients with periodontal disease; intervention: professional-mechanical plaque removal with subgingival air-polishing; comparison: ultrasonic subgingival professional mechanical-plaque removal; outcome: effective control of subgingival biofilm deposits.)

### 2.2. Search Strategy

To conduct this review, the authors (two independent researchers, M.S. and D.N.G.) performed an electronic search of relevant scientific databases using PubMed, Web of Science, and Scopus, limiting results to papers published between 1990 and 2022. The search, performed from January to February 2023, was conducted using keywords, such as “periodontitis”, “periimplantitis”, “subgingival air polishing”, “glycine powder air-polishing”, “GPAP”, “trehalose powder air-polishing”, “TPAP”, “erythritol powder air-polishing”, “EPAP”, and “air polishing devices”. (Table 1) The papers included for future analysis included randomized clinical trials (RCTs) or controlled clinical trials (CCTs), clinical trials, transversal and cohort studies, case reports, and meta-analyses. 

### 2.3. Exclusion Criteria for Studies

Some articles were excluded based on the following criteria: studies with outcomes and conclusions that were not relevant to the main goal of this review; studies with limited follow-up periods; studies that did not involve the type of interventions being investigated in this review; monographs; letters to the editor, due to the low grade of evidence or absence of a peer-reviewing process. 

### 2.4. Information Extraction and Review Structuring

The risk of bias was appreciated using the JADAD modified scale, with only papers scoring a minimum of grade 4 being further processed. After carefully selecting relevant articles, the information was extracted (by two independent researchers, M.S. and D.N.G., and a mediator, F.B.) and then organized into three parts for this review. The first part included studies that compared the efficiency of air-polishing powders to ultrasonic root smoothing, in addition to evaluating the abrasiveness of air-polishing powders on tooth tissues. The second part focused on the efficiency of various types of air-polishing powders for controlling subgingival biofilm formation on both tooth surfaces and implants. Finally, the third part examined the limitations of using subgingival air-polishing alone or in combination with other therapies, such as SRP and ultrasonic devices. The registration of the review was not possible, as data extraction and review structuring had already been initiated at the time of the attempted registration. 

## 3. Results

### 3.1. Basics of Periodontal Therapy

#### 3.1.1. Patient Motivation and Education

The initial stage of cause-related therapy involves educating patients about the primary etiological factor of periodontal disease: the accumulation of plaque both above and below the gumline [24]. This education is essential for enhancing patients’ comprehension of the disease and empowering them to effectively manage and prevent it [25,26]. Enhancing oral care practices at home holds significant importance in the prevention of periodontal disease, successful periodontal therapy, and the sustained maintenance of positive outcomes. Emphasizing the significance of meticulous home maintenance should remain a central focus throughout all stages of periodontal therapy [27]. 

#### 3.1.2. Professional Mechanical Plaque Removal

Professional mechanical plaque removal, as part of the secondary prevention of periodontitis (PMPR+), entails the regular removal of the subgingival plaque and calculus, accompanied by subgingival debridement down to the depth of the sulcus/pocket [28]. This procedure constitutes a crucial element of supportive periodontal therapy (SPT), which should encompass various components: an evaluation of oral hygiene practices, motivational and instructional guidance, counseling for smoking cessation, management of co-existing health conditions, and promotion of healthy lifestyles [28,29]. Furthermore, a comprehensive periodontal examination should be performed to detect early indications of deepening pockets (pocket depth ≥ 5 mm) that necessitate active periodontal intervention [30].

#### 3.1.3. Adjunctive Procedures

Scaling and root planing (SRP) are performed in areas where the periodontal probing depth measures 5 mm or more [28]. In conjunction with SRP, active carious lesions should be addressed, irreparable teeth should be extracted, and local contributing factors need to be managed [31,32]. 

As an adjunct to SRP for patients with moderate-to-severe periodontitis, systemic antibiotics can be employed [28,33]. The benefits of antibiotic use encompass decreased bleeding upon probing, a lower prevalence of lingering periodontal pockets, and the enhanced closure of such pockets [34]. The most notable advantage was evident with amoxicillin and metronidazole, administered at the highest effective dosage for the shortest feasible duration to mitigate the risk of antibiotic resistance. It is important to note that there are insufficient data to establish the superiority of any specific dosing regimen [35].

#### 3.1.4. Periodontal Reevaluation

Following the initial scaling and root planing, a re-evaluation should be conducted within four to six weeks to assess the extent of improvement [28]. This process entails updating the periodontal charting and comparing it to the original charting. Additionally, it is crucial to assess the patient’s adherence to the prescribed at-home oral care routine. In cases of relatively shallow probing depths (1–5 mm), non-surgical interventions, including periodic periodontal maintenance therapy, repeated root planing as required, and consistent encouragement of home care, may be considered [36].

However, for sites with deeper probing depths (≥6 mm), the effectiveness of subgingival calculus removal diminishes, and surgical periodontal therapy might be recommended. It is important to carefully evaluate each case to determine the most appropriate course of action for optimal periodontal health [36].

### 3.2. Subgingival Use of Air-Polishing Powders

#### 3.2.1. Effectiveness of Subgingival Air-Polishing

In a study conducted by Wenzler et al., subgingival air-polishing powders based on glycine and trehalose were found to possess antimicrobial properties capable of reducing periopathogenic bacteria, such as *Porphyromonas gingivalis* and *Tannerella forsythia* [37]. Comparing air-polishing using the trehalose powder to sonic scalers, a study by Kruse et al. revealed that it was equally effective while causing less discomfort and presenting a lower risk of damaging the tooth structure [38].

For the removal of biofilm in patients with periodontal pockets up to 5 mm, low-abrasive glycine powder can prove effective when used in conjunction with hand instruments or scalers. This treatment is considered comfortable and safe not only for periodontitis and peri-implantitis, but also in the fields of operative dentistry and orthodontics [39,40]. While this article primarily focuses on the air-polishing powder, it gives comparatively less emphasis on the device type. However, Kruse et al. cast doubt on the efficacy of trehalose powder in effectively removing subgingival biofilm, as their study found it had no impact on the microbiome of the regrown biofilm [38]. In a study by Sekino et al., it was observed that inflammation could still persist even in cases of supportive periodontal therapy when air-polishing with glycine powder and a special subgingival application nozzle was employed at 30-day intervals [41]. 

Regarding peri-implantitis, treatment with glycine air-polishing (GPAP) can be effective, although Jiang and Tong suggest that the improvement might be short-term [42]. Jing et al. concluded that subgingival glycine air-polishing, ultrasonic scaling, and 0.12% chlorhexidine rinsing were equally effective in treating early peri-implant diseases, with early treatment potentially being more effective in controlling inflammation [43]. Using air-polishing as the primary treatment without combining it with scaling and root planing in cases with a probing depth ≥ 6 mm may yield a successful short-term gain in the clinical attachment level (CAL), according to Schlagenhauf et al. [44]. Petersilka et al. noted that using GPAP alone does not improve the clinical outcome of periodontal maintenance compared to mechanical plaque removal [45]. Furthermore, using GPAP to address furcation defects appears to be contraindicated [45].

Zhao et al. indicated that air-polishing with 65 μm glycine powder is comparable to ultrasonic scaling combined with polishing paste in terms of clinical effects, but it should be limited to patients with shallow pockets and no visible tooth calculus [46]. Petersilka et al. demonstrated that the low-abrasive air-polishing powder is superior to curettes for removing subgingival plaque at interdental locations with probing depths up to 5 mm during periodontal maintenance therapy [40]. According to Laleman et al., no specific treatment demonstrates a preferable therapeutic advantages or significant microbiological variations, but methods, like laser therapy, photodynamic therapy, or air-polishing, cause less discomfort compared to hand- and/or (ultra)sonic instrumentation [47]. 

Bühler et al. suggested that powders containing glycine lead to less discomfort when performing both supra- and subgingival air-polishing as part of non-surgical periodontal therapy [48]. Petersilka et al. found that the innovative low-abrasive air-polishing powder is more effective than curettes for removing subgingival plaque from pockets that are 3–5 mm deep during supportive periodontal therapy and provides higher patient comfort [39]. Finally, according to Wennström et al., air-polishing was perceived as less uncomfortable during treatment compared to ultrasonic debridement [49]. Both methods significantly reduced BOP, PPD, and attachment loss at two months after treatment [49].

According to Lu et al., supragingival glycine air-polishing consistently proves effective in removing dental plaque biofilm during the maintenance phase, suggesting that a three-month interval may be appropriate for pockets no deeper than 5 mm [50]. Seidel et al. found that while powered scalers used in conventional mechanical debridement were the most efficient, air-polishing was faster [51]. The findings from Zhu et al. suggest that GPAP may have the potential to replace conventional treatments for gingival inflammation due to its potential for quicker treatment and reduced discomfort [52]. However, higher-quality studies are still necessary to comprehensively assess its effects [52].

The study conducted by Kargas et al. did not establish the superiority of GPAP over SRP or subgingival ultrasonic scaling as sole treatments, based on clinical or microbiological data [53]. Furthermore, research by Petersilka et al. provides additional evidence of the safety of GPAP, demonstrating that it results in less gingival erosion than sodium bicarbonate air-polishing (SBAP) or hand instrumentation [54]. Zhang et al. found that local periodontal therapy has a short-term impact on blood microbiota stability. Full-mouth SRP followed by adjunctive GPAP appears to be a potential method to reduce bacterial entry into the bloodstream during the procedure [55]. While SRP is effective in treating halitosis and periodontitis, using GPAP with mechanical instruments does not improve periodontal or halitosis characteristics, as reported by Caygur et al. [56].

In an in vitro investigation, Poornima et al. determined that hand root surface smoothing with curettes is more effective than ultrasonic root smoothing [57]. The addition of GPAP for 5 s to hand scaling or ultrasonic scaling during periodontal maintenance therapy improves the smoothness of the root surface. Both ultrasonic scaling (US) and air-polishing (AP) treatments effectively reduce the pathogenicity of the subgingival microbiome by lowering microbial diversity, decreasing the proportion of microbiota associated with periodontitis, and inhibiting pathogenic metabolism [57]. Lu et al. reported that this contributes to maintaining a balanced subgingival community and a stable periodontal state over a three-month maintenance period [58]. According to Flemmig et al., GPAP is more effective than SRP at removing subgingival biofilm in moderate-to-deep periodontal pockets [59]. Full-mouth GPAP is well-tolerated and can lead to a positive change in the oral microbiota [59].

#### 3.2.2. Differences in the Type of Powder

According to a study by Jentsch et al., the use of erythritol air-polishing powder as an adjunct to subgingival instrumentation does not supplementarily reduce bleeding on probing (BOP) [60]. However, it may exert positive effects, such as reducing the frequency of residual periodontal pockets with a probing depth of 5 mm [60]. Resnik et al. discovered that during the initial phase of non-surgical periodontal therapy, subgingival airflow therapy combined with an erythritol powder air-polishing device (EPAP) might benefit patients with initially deep pockets (probing pocket depth of 5.5 mm) [61]. Both treatment methods significantly reduced periodontitis-related bacterial species, as well as BoP, PPD, and the relative attachment level. No significant differences were found between the various treatment methods [61].

Another argument supporting the effectiveness of less traumatic air-polishing technology with new, less abrasive powders is presented by Moëne et al. [62]. However, they noted that there is limited short-term research on this topic. In contrast, Mensi et al. [63] found no significant additional advantage for patients with periodontitis at stages 3–4. According to Flemmig et al., subgingival biofilm in periodontal pockets with an APD ≤ 3 mm can be effectively removed by GPAP for 5 s per surface [15]. Hu et al. suggest that subgingival air-polishing and conventional manual scaling can improve supportive periodontal therapy in teeth with probing depths of 3 to 6 mm, but there is no discernible difference between the two modes [64].

Air-polishing with erythritol may cause a slight loss of dentin, influenced by the environment, as indicated by Kröger et al. [65]. Applying erythritol powder with an air-polishing tool seems to be a promising method for repeatedly instrumenting residual pockets during SPT, according to Hägi et al. [66]. Ulvik et al. suggest that both erythritol air-polishing and traditional mechanical debridement contribute to therapeutic improvements in mandibular furcations [67]. However, at the 6-month follow-up, traditional debridement was found to be more effective in terms of the clinical attachment level. Nonetheless, patients reported that they found the erythritol air-polishing device to be the most comfortable treatment [67].

Zhang et al. found that full-mouth SRP with or without GPAP had comparable effects on clinical, inflammatory, and microbiological outcomes in treating untreated periodontitis [68]. Similarly, Hägi et al. reported that subgingival EPAP is safe and provides similar clinical and microbiological results to SRP [69]. On the other hand, Simon et al. demonstrated that GPAP causes less gingival erosion than SBAP or ultrasonic instruments and improves plaque and gingival index scores [70].

In vitro studies have shown that subgingival air-polishing powders can affect wound healing, cell viability, morphology, and proliferation. The cytotoxicity of erythritol/clorhexidine (CHX) powder is primarily caused by the CHX component and can directly affect gingival fibroblasts [71]. Different polyols have varying impacts on dental health, with erythritol showing more effectiveness than sorbitol and xylitol in enhancing oral health [72]. Recurrent subgingival debridement using APDs during SPT produced similar clinical outcomes, but better patient comfort compared to traditional therapies, though there is insufficient evidence to support their superiority for implant maintenance [73]. An in vitro study [74] revealed comparable root surface loss with glycine powders, but spray patterns and tip apertures can influence the powder performance between units. Cementum loss may be higher than that with traditional biofilm removal techniques, but more research is needed. Finally, according to Bains et al. [75], GPAP causes less gingival erosion than manual instrumentation or sodium bicarbonate air-polishing, but selective polishing data for teeth remain convincing despite advancements in the polishing technology.

According to Schulz et al., all therapeutic treatments tested, including full-mouth scaling (FMS), full-mouth disinfection (FMD), and adjuvant erythritol air-polishing (FMDAP), were effective in reducing harmful bacteria in the short term [76]. However, after six months, there was a decline in the microbiological profile, and clinical outcomes either improved or remained stable. Studying subgingival bacteria may aid in evaluating periodontal treatment and implementing personalized therapy. Air-polishing therapy is the most effective treatment for cleaning implant surfaces during biofilm removal, and a 45 s treatment period significantly improves its performance, as stated by Mensi et al. [77].

#### 3.2.3. Cellular and Tissular Impact

A systematic review conducted by Nascimento et al. found that air-polishing and hand or ultrasonic instruments have similar effectiveness in controlling biofilm formation and reducing periodontal inflammation [78]. This suggests that air-polishing can be considered an alternative for biofilm control [79]. Another systematic review suggests that there is no significant difference in the clinical outcomes of subgingival air-polishing and ultrasonic debridement [80]. However, the review acknowledges the limited evidence and calls for further long-term studies 80]. Moene et al. found that while a new subgingival air-polishing device was more time-saving and acceptable for patients, it did not have any microbiological advantages over traditional scaling and root planing (SRP) treatment [62].

Cannabidiol (CBD)-supplemented polishing powder, according to Stahl et al., can assist in the efficient removal and death of dental plaque bacteria during the polishing procedure and can be added as an enhancing supplement to currently available polishing powders [81]. Di Tinco et al. found that both powders are suitable for boosting the biocompatibility of titanium implants, since they exhibit excellent in vitro cleaning potential in the early stages and have no detrimental effects on human dental pulp stem cells’ osteogenic differentiation process [82]. 

In vitro research by Weusmann et al. suggests that powders for air-polishing tools that can be applied subgingivally may control cytokine expression, cell survival, and proliferation [83]. The study indicates that these powders may affect growth factors through direct cell actions, and trehalose seems to be more inert than glycine powder. Even though some patients with periodontitis can regain their periodontal health, the majority of them will always be at risk of developing the condition [84]. Accurately assessing a patient’s periodontal risk will allow for the proper monitoring of those who have undergone appropriate periodontal disease treatment.

Air-polishing was found to be superior to ultrasonic scalers in preserving cementum, while hand curettes were the most effective at removing cementum [85]. In a randomized controlled trial, periodontal therapy with adjunctive photodynamic therapy (aPDT) showed improved clinical outcomes and decreased levels of pathogenic bacteria in the subgingival biofilm when compared to scaling and root planing alone [86]. The effect was noted to be more pronounced in patients with deeper pockets and higher levels of initial bacterial colonization [86]. According to Dalvi et al., photobiomodulation therapy (PBMT) has shown promise as an adjunctive therapy for periodontitis. PBMT can reduce inflammation, promote tissue healing, and decrease the levels of harmful bacteria [87]. However, more high-quality clinical studies are required to confirm its effectiveness and determine the optimal parameters for treatment [87].

In a study by Persson et al., the air-abrasive group showed a reduction in *P. aeruginosa*, *S. aureus*, and *S. anaerobius*, while the laser group saw a decrease in *Fusobacterium* spp. after 1 month of treatment [88]. However, neither approach resulted in a significant decrease in bacterial numbers over a 6-month period, and there were no substantial clinical improvements observed [88]. McCollum et al. used a digitizer and software to calculate the percentage of the total abutment surface area covered by plaque, distinguishing between subgingival and supragingival plaque [89]. The study found that the air-powder abrasive had a total mean percent plaque surface area of 52.06%, while the plastic scalers had 55.29% [89,90].

## 4. Future Perspectives

While the current body of research provides valuable insights, there remain areas that warrant further exploration. Long-term studies, standardized protocols, and investigations into patient-centered outcomes are crucial to establishing the sustained efficacy and patient preference for air-polishing. Additionally, research into the effects of air-polishing on the oral microbiome, implant maintenance, and its potential combination with other therapies will contribute to a comprehensive understanding of its applications and benefits (Table 2). 

As the dental community seeks innovative and patient-friendly approaches to periodontal care, air-polishing emerges as a promising tool that aligns with these goals. With continued research, the refinement of techniques, and collaboration among researchers, practitioners, and regulatory bodies, the future of air-polishing in periodontal therapy holds the potential to enhance patient outcomes and contribute to the advancement of modern periodontology. This could be achieved through the following:

1. Long-term comparative studies: conducting more long-term comparative studies to evaluate the sustained effects of air-polishing versus traditional methods, like ultrasonic scaling or hand instrumentation. This can provide a better understanding of the extended benefits and potential drawbacks of air-polishing over time.
2. Optimal parameters and techniques: further investigation into optimal air-polishing parameters, such as powder types, particle sizes, pressure, and angles, to maximize biofilm removal efficacy while minimizing any potential adverse effects on tooth surfaces or soft tissues.
3. Standardization of protocols: development of standardized protocols and guidelines for incorporating air-polishing into periodontal therapy, considering factors, like patient-specific conditions, disease severity, and treatment intervals. This can ensure consistency and reproducibility across different clinical settings.
4. Patient-centered outcomes: exploration of patient-centered outcomes beyond clinical parameters, such as patient comfort, satisfaction, and quality of life, to assess the overall acceptability and preference for air-polishing compared to traditional methods (such as the Dental Visit Satisfaction Scale, Dental Satisfaction Questionnaire or Patient Assessment Questionnaire) [91].
5. Subgingival applications: continued research into the safety, efficacy, and long-term effects of subgingival air-polishing, especially in deeper pockets and challenging-to-reach areas. This may involve investigating different nozzle designs, powder formulations, and application techniques.
6. Combination therapies: investigating the potential synergistic effects of combining air-polishing with other adjunctive therapies, such as photodynamic therapy, laser therapy, or antimicrobial agents, to enhance biofilm removal and overall treatment outcomes.
7. Impact on the microbiome: further studies on the impact of air-polishing on the oral microbiome, both short-term and long-term, to better understand how it influences the balance of beneficial and pathogenic microorganisms in the periodontal environment.
8. Implant maintenance: exploring the application of air-polishing for implant maintenance, including its effects on peri-implant health, biofilm removal from implant surfaces, and its potential role in preventing peri-implant diseases.
9. Personalized treatment approaches: Research into the development of personalized treatment approaches that consider individual patient characteristics, genetics, and the microbiome composition to tailor air-polishing techniques and protocols for optimized outcomes.
10. Education and training: enhanced education and training for dental professionals on the proper use of air-polishing equipment, techniques, and patient selection, ensuring its safe and effective integration into clinical practice.
11. Economic considerations: investigating the cost-effectiveness of air-polishing compared to traditional methods, considering factors, such as reduced chair time, patient satisfaction, and long-term maintenance requirements.
12. Regulatory approval and guidelines: collaborating with regulatory bodies and dental associations to establish clear guidelines and recommendations for the use of air-polishing in periodontal therapy, ensuring patient safety and standardized practices.

As research and technology continue to advance, these perspectives can help shape the future of air-polishing in periodontal therapy, leading to improved patient care and enhanced treatment outcomes [92,93]. 

**Table 2:** Synopsis of reviewed papers in alphabetical order (GPAP = glycine powder air-polishing, EPAP = erythritol powder air-polishing, TPAP = trehalose powder air-polishing).

Study	Air-Polishing Powder	Air-Polishing Device	On Teeth/On Implants	Findings
(Bains, et al., 2009) Review, India [75]	GPAP	-	Teeth	GPAP, which has recently been shown to remove subgingival biofilm, causes less gingival erosion than manual instrumentation or NaHCO(3) air-polishing. Despite the development of recent polishing advancements, data supporting the selective polishing of teeth remains persuasive.
(Bozbay, et al., 2018) Randomized Controlled Trial, Italy [85]	-	-	Teeth	Air-polishing was superior than ultrasonic scaler devices in cementum preservation, whereas hand curettes were the most efficient cementum-removal tools.
(Bühler, et al., 2016) Systematic Review, Switzerland [48]	GPAP	Electro Medical Systems (EMS)^®^ Nyon, Switzerland	Teeth	When performing supra- and subgingival air-polishing as part of non-surgical periodontal therapy, using powders containing glycine, there seems to be less discomfort.
(Caygur, et al., 2017) Randomized Controlled Trial, Turkey [56]	GPAP	Air-Flow Perio^®^ Powder; Electro Medical Systems^®^ Nyon, Switzerland	Teeth	Treatment of halitosis and periodontitis with SRP is successful. However, employing GPAP in conjunction with mechanical instruments does not improve periodontal or halitosis characteristics.
(de Cock, et al., 2016) Review, Belgium [72]	EPAP	-	Teeth	The evidence in the research shows that erythritol is more effective in preserving and enhancing oral health than sorbitol and xylitol.
(Di Tinco, et al., 2021) In vitro, Italy [82]	GPAP TPAP	-	Implant	Both powders are suitable for boosting the biocompatibility of titanium implants since they both exhibit excellent in vitro cleaning potential in the early stages and have no detrimental impacts on hDPSCs’ osteogenic differentiation process.
(Divnic-Resnik, et al., 2022) Randomized Controlled Trial, Australia [61]	EPAP	Air-flow Plus^®^, EMS, Electro Medical Systems, Nyon, Switzerland	Teeth	Initially deep pockets (PPD 5.5 mm) may benefit from subgingival airflow therapy combined with EPAP during the first phase of non-surgical periodontal therapy.
(Flemmig, et al., 2012) Randomized Controlled Trial, USA [59]	GPAP	EMS Air-Flow^®^, Electro Medical Systems EMS SA, Nyon, Switzerland	Teeth	Most of the subgingival biofilm in periodontal pockets with an APD ≤3 mm can be effectively removed by GPAP for 5 s per surface.
(Flemmig, et al., 2015) Randomized Controlled Trial, USA [15]	GPAP	AIR-FLOW PERIO Powder^®^, E.M.S. Electro Medical Systems, Nyon, Switzerland. AIR-FLOW Master^®^, E.M.S. Electro Medical Systems. AIR-FLOW PERIO^®^, E.M.S. Electro Medical Systems.	Teeth	SubGPAP is more effective than SRP at removing subgingival biofilm in moderate-to-deep periodontal pockets. Furthermore, full-mouth GPAP seems to be well tolerated and may lead to a positive change in the oral microbiota.
(Hägi, et al., 2013) Randomized Controlled Trial, Switzerland [66]	EPAP	-	Teeth	A promising method for repeatedly instrumenting leftover pockets during SPT is the erythritol powder applied with an air-polishing tool.
(Hägi, et al., 2015) Randomized Controlled Trial, Switzerland [69]	EPAP GPAP	Air-Flow Master Piezon^®^, EMS, Nyon, Switzerland Air-Flow Master Piezon^®^, EMS; Air-Flow Powder Plus^®^ 0.3% CHX, EMS, Nyon, Switzerland	Teeth	Applied ultrasonication and air-polishing with erythritol, as opposed to hand instrumentation, reduce material loss and produce a smooth surface with almost little residual biofilm, which encourages the reattachment of PDL fibroblasts. The subgingival application of EPAP using air-polishing equipment may be considered secure and may provide clinical and microbiologic results that are similar to those of SRP.
(Herr, et al., 2017) In vitro, USA [74]	GPAP	In vitro	Teeth	An evaluation of glycine powders revealed comparable root surface loss. Spray patterns and tip apertures may play a role in variations in powder performance between units. Cementum loss may be larger than that experienced with traditional biofilm-removal techniques, such as curets and ultrasonic scalers, although further investigation is required to confirm this.
(Hu, et al., 2015) Randomized Controlled Trial, China [64]	-	-	Teeth	In teeth with probing depths of 3 to 6 mm, supportive periodontal therapy can be improved clinically with subgingival air-polishing and conventional manual scaling.
(Jentsch, et al., 2020) Randomized Controlled Trial, Germany [60]	EPAP	Hu-Friedy^®^ Manufacturing Co., Chicago, IL, USA and Dentsply Sirona, Bensheim, Germany	Teeth	Compared to subgingival instrumentation alone, decreasing the probing depth, as indicated by the frequency of residual periodontal pockets with PD 5 mm.
(Jiang & Tong, 2019) Clinical Trial, Chi [42]	GPAP	-	Implant	Treatment with air-polishing can be effective, although the improvement is short-term.
(Jing, et al., 2017) Randomized Controlled Trial, China [43]	GPAP	-	Implant	Based on patients with early peri-implant diseases, the effectiveness of subgingival glycine air-polishing, ultrasonic scaling, and 0.12% chlorhexidine rinsing is similar. The early peri-implant inflammation may be more effectively controlled by the earlier treatment.
(Kargas, et al., 2015) Randomized Controlled Trial, Greece [53]	GPAP	-	Teeth	The study does not demonstrate the superiority of GPAP over SRP or subgingival ultrasonic scaling when used as the sole treatment, based on clinical or microbiological data.
(Kröger, et al., 2020) In vitro, Germany [65]	EPAP	-	Teeth	Depending on the distance, pressure, and angulation of the spray jet to the surface, a slight loss of dentin may occur after air-polishing with erythritol. The quantity of dentin loss is influenced by the environment.
(Kruse, et al., 2019) Randomized Controlled Trial, Germany [38]	TPAP	(1) Lunos^®^ Prophylaxis Powder Perio Combi, DÜRR DENTAL, Bietigheim-Bissingen Germany (2) Perio-Flow^®^ handpiece with Perio-Flow^®^ Nozzle EMS, Nyon, Switzerland (3) Sonic Flex, KaVo, Charlotte, NC, USA	Teeth	Air-polishing to sonic scalers appear to provide an equivalent clinical result, with air-polishing causing less discomfort and less risk of damaging the tooth.
(Laleman, et al., 2017) Review, Belgium [47]	GPAP	-	Teeth	Regarding the therapeutic advantages or microbiological variations, none of these treatments appear to be preferable to any other. However, when compared to hand- and/or (ultra)sonic instrumentation, less treatment discomfort is recorded when employing laser, photodynamic therapy, or air-polishing.
(Lu, et al., 2018) Randomized Controlled Trial, China [50]	GPAP	Air-Flow Polishing Soft^®^; Air-Flow handy2^®^ Air-Flow Masters^®^ (EMS, Nyon, Switzerland)	Teeth	The removal of subgingival dental plaque biofilm via supragingival glycine air-polishing was consistently effective during the maintenance phase, and three months may be an appropriate maintenance interval for pockets that are no deeper than 5 mm.
(Lu, et al., 2019) Randomized Controlled Trial, China [58]		-	Teeth	By reducing microbial diversity, the proportion of microbiota associated with periodontitis, and pathogenic metabolism, treatment with US or AP successfully lowered the pathogenicity of the subgingival microbiome. Over a single maintenance period of three months, it assisted in maintaining a balanced subgingival community and stable periodontal state.
(McCollum, et al., 1992) Comparative Study, USA [89]	flour of pumice	-	Teeth	The percentage of the total abutment surface area that the plaque covered was calculated using a digitizer and software. Subgingival and supragingival plaque were clearly distinguished from one another. Between 52.06% for the air-powder abrasive and 55.29% for the plastic scalers, the total mean percent plaque surface area was measured
(Mensi, et al., 2020) In vitro, Italy [77]	EPAP GPAP	-	Implant	The best treatment for cleaning the implant surface in the ink removal among the four options is air-polishing therapy. Furthermore, air-polishing performed significantly better when the treatment period was increased to 45 s
(Mensi, et al., 2020) Randomized Controlled Trial, Italy [63]	EPAP	Airflow prophylaxis Master^®^, EMS, Nyon, Switzerland	Teeth	There is no significant additional advantage for periodontitis patients at stages 3–4.
(Mensi, et al., 2017) Clinical Trial, Italy [79]	-	-	Teeth	Clinical outcomes produced by the OSFMI treatment were comparable to those attained with conventional SRP. It is advised for researchers to explore this approach in randomized clinical trials with longer observation times.
(Moene, et al., 2010) Randomized Controlled Trial, Switzerland [62]	GPAP	EMS^®^, Nyon, Switzerland	Teeth	The use of a new device for subgingival air-polishing was time-saving for patients and also more acceptable. Even so, it was not microbiologically superior to traditional SRP.
(Nascimento, et al., 2021) Review, Denmark [78]	GPAP (10) EPAP (2) TPAP (1)	EMS^®^ Air Flow S1, Nyon, Switzerland	Teeth	Air-polishing is an alternative for biofilm control. The comparation between air-polishing and hand or ultrasonic instruments can be equal in controlling biofilm formation and also reducing periodontal inflammation.
(Persson, et al., 2011) Randomized Controlled Trial, Sweden [88]	-	-	Teeth	At 1 month, the air-abrasive group had a decrease in P. aeruginosa, S. aureus, and S. anaerobius, whereas the laser group saw a decrease in Fusobacterium spp. Data collected over a six-month period showed that neither approach reduced bacterial numbers. Clinical advancements were not substantial.
(Petersilka, 2011) Review, Germany [92]	GPAP	EMS Airflow S1^®^ and Easy Jet Pro^®^, Mectron, Munich, Germany	Teeth and implants	For biofilm removal, in periodontal patients with pockets up to 5 mm, low-abrasive glycine powder can be effective but is also needed a hand instrument or scaler. It is considered a comfortable and safe choice for the treatment of both periodontitis and peri-implantitis and it can be useful in operative dentistry, as well as in orthodontics.
(Petersilka, et al., 2003) Clinical Trial, Germany [39]	low-abrasive air-polishing powders	EMS Air Flow S1^®^, EMS, Nyon, Switzerland.	Teeth	When it comes to removing subgingival plaque at interdental locations with up to 5 mm of probing depth in PMT, the innovative low-abrasive air-polishing powder is superior to curets.
(Petersilka, et al., 2003) Clinical Trial, Germany [40]	Sodium bicarbonate Powder D	EMS Air Flow S1^®^, EMS, Nyon, Switzerland	Teeth	The innovative low-abrasive air-polishing powder provides higher patient comfort and is more effective than curettes in removing subgingival plaque from pockets that are 3–5 mm deep during supportive periodontal therapy.
(Petersilka, et al., 2008) Randomized Controlled Trial, Germany [54]	GPAP	EMS AirFlow^®^ Powder, EMS, Nyon, Switzerland	Teeth	The research supported the safety of this debridement method by showing that GPAP causes less gingival erosion than SBAP or hand instrumentation.
(Petersilka, et al., 2020) Clinical Trial, Germany [45]	GPAP	-	Teeth Furcation defects	The use of GPAP alone to address furcation defects is contraindicated.
(Poornima, et al., 2019) Comparative Study, India [57]	GPAP	In vitro	Teeth	In this in vitro investigation, hand root surface smoothing with curettes was more effective than ultrasonic root smoothing. During periodontal maintenance therapy, the addition of GPAP for 5 s to hand scaling or ultrasonic scaling increased the smoothness of the root surface.
(Schlagenhauf, et al., 2021) Randomized Controlled Trial, Germany [44]	EPAP	AIRFLOW Prophylaxis Master^®^, EMS, Nyon, Switzerland	Teeth	Ιn cases with a probing depth ≥ 6 mm, the results are successful only for the short-term gain of CAL.
(Schulz, et al., 2022) Randomized Controlled Trial, Germany [76]	EPAP	E.M.S.^®^ Electro Medical Systems, Nyon/Switzerland	Teeth	The reduction in harmful germs in the FMS, FMD, and FMDAP groups was linked to the effectiveness of all therapeutic therapies tested three months later. But after six months, they noticed a decline in the microbiological profile and either more improvement or some standstill in the clinical outcomes. A good periodontal treatment evaluation and personalized therapy implementation may be aided by research into the subgingival bacteria.
(Seidel, et al., 2021) Clinical Trial, Germany [51]	GPAP EPAP	PERIOFLOW^®^ handpiece, EMS, Nyon, Switzerland Proxeo ultra^®^, W&H, Bürmoos, Austria AIR-FLOW PLUS^®^ powder, EMS, Nyon, Switzerland	Teeth	Powered scalers used in conventional mechanical debridement were most efficient, but air-polishing was faster.
(Sekino, et al., 2020) Randomized Controlled Trial, Japan [41]	GPAP	Perio-Flow^®^ Nozzle, EMS, Nyon, Switzerland	Teeth	Inflammation can be present in cases of supportive periodontal therapy, using un special nozzle for subgingival applications with air-polishing.
(Simon, et al., 2015) Randomized Controlled Trial, India [70]	GPAP	EMS—Air Flow classic Powder^®^ Nyon, Switzerland; Dentsply ProphyJet^®^, Dentsply, York, PA, USA	Teeth	GPAP induces histologically less gingival erosion than SBAP or ultrasonic instruments and leads to clinically substantial improvements in plaque and gingival index scores.
(Stahl, et al., 2020) Clinical Trial, Belgium [81]	+CBD	In vitro	Teeth	In addition to being added as an enhancing supplement to the currently available polishing powders, the CBD (cannabidiol)-supplemented polishing powder can assist in the efficient removal and death of dental plaque bacteria during the polishing procedure.
(Tan, et al., 2022) Meta-analysis, Malaysia [73]	APDs in SPT	-	Implant	There is insufficient evidence to conclude that APDs are superior to conventional therapies for implant maintenance when they are applied repeatedly.
(Ulvik, et al., 2021) Randomized Controlled Trial, Norway [67]	EPAP	Air-flow powder plus^®^, EMS, Nyon, Switzerland Air-Flow Master^®^, EMS, Nyon, Switzerland	Teeth Furcation defects	Erythritol air-polishing and traditional mechanical debridement both assist in therapeutic advancements to the of mandibular furcations. However, a significant difference in the clinical attachment level across treatments was found at 6 months, favoring traditional debridement. The patients thought that the erythritol air-polishing device was the most comfortable treatment.
(Wennström, et al., 2011) Randomized Controlled Trial, Sweden [49]	GPAP	Air-Flows Perio Powder^®^, EMS, Nyon, Switzerland Air-Flow Masters^®^, EMS Perio-Flows Nozzle^®^, EMS EMS Piezon Masters^®^ 400, PerioSlim^®^ tip, EMS, Nyon, Switzerland	Teeth	Both treatment methods significantly decreased the number of periodontitis-related bacterial species both immediately and two days after treatment, in addition to significantly lowering the BoP, PPD, and relative attachment level at two months. At any of the examination periods, there were no statistically significant differences between the various treatment methods. Compared to ultrasonic debridement, air-polishing was perceived as less uncomfortable during treatment.
(Wenzler, et al., 2021) In vitro, Germany [37]	GPAP TPAP	Air-Flow Perio^®^ EMS, Nyon, Switzerland	Teeth	The subgingival air-polishing powders (glycine and trehalose) can, on the one hand, reduce periopathogenic bacteria, such as *Porphyromonas gingivalis* and *Tannerella forsythia*, and also provide an antimicrobial therapy approach.
(Weusmann, et al., 2021) In vitro, Germany [83]	GPAP TPAP	In vitro	Teeth	Powders for air-polishing tools that can be applied subgingivally can control cytokine expression, cell survival, and proliferation. This vitro research indicates that the aforementioned powders may affect HGF through direct cell actions. When compared to glycine powder, trehalose seems to be more inert.
(Weusmann, et al., 2022) In vitro, Germany [71]	EPAP+ CHX	In vitro	Teeth	The cytotoxic effect of erythritol/CHX powder is highly evident and primarily caused by the CHX component. These effects on fibroblasts are apparent and imply that powders applied subgingival have the ability to influence gingival fibroblasts directly.
(Zhang, et al., 2019) Review, China [80]	GPAP EPAP TPAP Sodium Bicarb.	-	Teeth	Neither subgingival air-polishing nor ultrasonic debridement seemed to have superior clinical results.
(Zhang, et al., 2021) Randomized Controlled Trial, China [55]	GPAP	Air-Flow Polishing^®^ Soft; EMS, Nyon, Switzerland Perio-Flow^®^ Nozzle^®^, EMS, Switzerland	Teeth	The microbiological effects of full-mouth SRP with and without GPAP in the treatment of untreated periodontitis were substantially comparable.
(Zhang, et al., 2021) Randomized Controlled Trial, China [68]	GPAP	Air-Flow^®^ Polishing Soft Powder; EMS, Nyon, Switzerland Perio-Flow^®^, Nozzle^®^, EMS, Switzerland	Teeth	The short-term effects of local periodontal therapy just disturb the stability of the blood microbiota. A possible method to minimize the entrance of bacteria into the bloodstream during the procedure is full-mouth SRP followed by adjunctive GPAP in the treatment of periodontitis.
(Zhao, et al., 2017) Randomized Controlled Trial, China [46]	GPAP	-	Teeth	Air-polishing with 65 μm glycine powder provides clinical effects that are comparable to those of ultrasonic scaling combined with polishing paste. Clinical indications should, however, only be used on patients who have shallow pockets and no visible tooth calculus.
(Zhu, et al., 2021) Meta-analysis, China [52]	GPAP	-	Teeth	The results of this study point to GPAP as a potential replacement for conventional treatments for gingival inflammation since it may do so more quickly and with less discomfort. Studies of a higher caliber are still required to evaluate the effects of GPAP.

## 5. Conclusions

In conclusion, the evolving field of air-polishing in periodontal therapy holds promise as an effective and less invasive approach for biofilm removal and periodontal health maintenance. A comprehensive review of the literature reveals a growing body of evidence supporting the efficacy of air-polishing in controlling biofilm formation, reducing periodontal inflammation, and contributing to overall periodontal health. Various studies have explored the use of different powders, equipment, and techniques, shedding light on the potential benefits and limitations of this innovative method.

Subgingival air-polishing has been shown to be comparable to traditional methods, such as ultrasonic scaling and hand instrumentation, in terms of clinical outcomes, suggesting that it can be considered a viable alternative or adjunctive therapy for managing periodontal conditions. Furthermore, advancements in powder formulations, nozzle designs, and application protocols continue to refine the practice of air-polishing, contributing to improved patient comfort and satisfaction during treatment.

## Figures and Tables

**Table 1:** Terms used in the research.

Database	Key Word Search
PUBMED	(“glycine powder, air-polishing”[Mesh]) AND “Periodontitis”[Mesh]; (“erythritol powder, air-polishing”[Mesh]) AND “Periodontal Diseases”[Mesh]; (“trehalose powder, air-polishing”[Mesh]) AND “Periodontal Status”[Mesh]; (“air-polishing, device”[Mesh]) AND “Periodontitis”[Mesh]; (“subgingival, air-polishing”[Mesh]) AND “Periimplantitis”[Mesh];
Web of Science	TS = (“subgingival air-polishing”) AND TS = (“Periodontal Diseases”); TS = (“subgingival air-polishing”) AND TS = (“Periodontitis”); TS = (“subgingival air-polishing”) AND TS = (“Periimplantitis”).
SCOPUS	ALL(“Subgingival air-polishing”) AND ALL(“Periodontal Diseases”); ALL(“Subgingival air-polishing”) AND ALL(“Periodontitis”); TITLE-ABS-KEY(“Subgingival air-polishing”) AND TITLE-ABS-KEY(“Periodontal Diseases”); TITLE-ABS-KEY(“Subgingival air-polishing”) AND TITLE-ABS-KEY(“Periimplantitis”).

## Data Availability

Not applicable.
